# Asymmetry of muscle co-activation during the two-armed kettlebell swing: insights into neuromuscular stability

**DOI:** 10.3389/fbioe.2025.1655248

**Published:** 2025-12-18

**Authors:** Khaled Abuwarda, Abdulazeem Alotaibi, Abdel-Rahman Akl

**Affiliations:** 1 Department of Physical Education and Kinesiology, College of Education, Qassim University, Qassim, Saudi Arabia; 2 Faculty of Sports Science-Abo Qir, Alexandria University, Alexandria, Egypt

**Keywords:** bilateral symmetry, injury risk assessment, motion analysis, physical activity, strength training, wearable sensors

## Abstract

**Introduction:**

Bilateral asymmetry reflects strength or functional differences between dominant and non-dominant limbs, which can influence performance. Investigating asymmetry and muscle co-activation during the two-armed kettlebell swing may clarify its role in performance enhancement and injury risk. This study specifically examined co-activation and asymmetry in shoulder and trunk muscles during the exercise.

**Method:**

Twenty-four participants (age: 23.9 ± 2.5 years; body mass: 82.8 ± 8.0 kg; height: 177.8 ± 6.5 cm) were included in the study. Surface electromyography signals were recorded using a wireless EMG system and data were collected bilaterally from twelve muscles (six muscles per side: anterior deltoid, posterior deltoid, erector spinae longissimus, erector spinae iliocostalis, external oblique, and rectus abdominis). Each participant completed two trials of the two-armed kettlebell swing, with at least five repetitions.

**Results:**

The results showed that the shoulder co-activation index significantly increased on both sides during the float, drop, and deceleration phases (Right side: F = 35.12; p < 0.001; Left side: F = 69.80; p < 0.001). Additionally, co-activation between the erector spinae and rectus abdominis, as well as between the erector spinae and external oblique muscles, was highest during the float and drop phases (Right side: F = 165.1; p < 0.001; Left side: F = 100.08; p < 0.001). The findings revealed some asymmetry in muscle co-activation, particularly during the float phase (22.39%). However, overall asymmetrical levels remained low during the more mechanically demanding phases (propulsion, drop, and deceleration).

**Conclusion:**

The study shows clear phase-dependent muscle activation patterns, with anterior deltoid and spinal extensors leading during propulsion, and greater posterior engagement and co-activation stabilizing the float, drop, and deceleration phases. A small asymmetry appeared mainly in the float phase, while the overall asymmetry index stayed low during the more demanding phases.

## 1 Introduction

Kettlebell (KB) training has received growing research attention since 2009, particularly for its impact on strength and conditioning (Busch et al., 2024). KBs are commonly used to improve muscular strength (Padovan et al., 2024) and are frequently incorporated into upper-extremity training programs. Their versatility makes them valuable for enhancing strength, improving lifting performance, supporting rehabilitation, and potentially preventing injuries by promoting muscular balance and joint stability (Stagi et al., 2023; Busch et al., 2024; Padovan et al., 2024).

Understanding muscle activation during KB exercises offers insight into their roles in injury prevention and performance enhancement (Salem et al., 2021; Padovan et al., 2024). Targeting shoulder muscles prone to imbalance or injury may be particularly effective in preventive programs (Busch et al., 2024). In athletic contexts, the standing variation of KB exercises is often preferred, as it activates not only the shoulder muscles but also core stabilizers such as the rectus abdominis, external obliques, and erector spinae (Padovan et al., 2024). Surface electromyography (sEMG) is commonly used to assess muscle activation, providing electrophysiological data useful for evaluating neuromuscular function and supporting neurorehabilitation (Bao et al., 2024). However, KB movements rely on complex sensorimotor integration, and the nonlinear characteristics of muscle activity limit the ability of traditional linear EMG measures such as amplitude to fully capture the underlying neuromuscular dynamics (Bao et al., 2024).

sEMG is commonly employed to estimate both muscle activation and co-activation patterns (Lee et al., 2017). Muscle co-activation is a central nervous system strategy used to simplify motor control and enhance joint stability during motor learning and movement regulation (Lee et al., 2017; Garcia-Vicencio et al., 2024). In particular, antagonist muscle co-activation is a critical neuromuscular mechanism for increasing joint stiffness and stability (DeVita and Hortobagyi, 2000; Pincivero et al., 2019). However, increased co-activation also necessitates greater agonist activation to achieve a given amount of net joint work (Waanders et al., 2021). The simultaneous contraction of antagonistic muscles can stabilize joints and improve upper limb control during tasks requiring high positional accuracy (Mari et al., 2014).

Unilateral sports such as tennis, rowing, and fencing often produce notable asymmetries due to their repetitive, one-sided movement patterns. These asymmetries contrast with the more balanced movement demands of bilateral sports such as swimming, walking, or gymnastics (Stagi et al., 2023; Herrera-Amante et al., 2025). Inter-limb strength asymmetry has been assessed using various methods, including isokinetic dynamometry (Menzel et al., 2013), unilateral and bilateral isometric mid-thigh pulls (Bailey et al., 2013), squat exercises (Bishop et al., 2021), single-leg countermovement jumps (unilateral and bilateral) (Bishop et al., 2019), drop jumps (Bishop et al., 2022) horizontal jumps (Hewit et al., 2012), deadlifts (Whittal et al., 2020). More recently, kettlebell exercises particularly the kettlebell swing have been examined as a dynamic task that challenges bilateral coordination while still allowing subtle inter-limb differences to emerge (Abuwarda and Akl, 2024).

Asymmetry typically refers to differences in strength or function between an individual’s dominant and non-dominant limbs, which may affect athletic performance (Ujaković and Šarabon, 2021; D'Hondt and Chapelle, 2024; Arboix-Alió et al., 2025). The growing interest in inter-limb asymmetry stems from its potential association with injury risk (Guan et al., 2021), and its relevance to injury prevention strategies (Herrera-Amante et al., 2025). Some studies suggest that moderate asymmetry in strength or muscle mass may provide a competitive edge in asymmetric sports. However, excessive imbalances may elevate the risk of injury (Šarabon et al., 2020; Parpa and Michaelides, 2022; Herrera-Amante et al., 2025). Herrera-Amante et al. (2025) highlighted the complex and multifactorial relationship between asymmetry and athletic performance, emphasizing the need for further research to understand how training affects symmetry and its impact on different sports disciplines. In the sports domain, the study of body symmetry is of critical importance, offering valuable insights into performance evaluation, physical development and maturation, injury prevention, training optimization, and equipment design (Herrera-Amante et al., 2025).

Surprisingly, no study to date has investigated muscle co-activation asymmetry as a critical neuromuscular mechanism for enhancing joint stiffness and stability (DeVita and Hortobagyi, 2000; Pincivero et al., 2019), This factor may influence athletic performance (Ujaković and Šarabon, 2021; D'Hondt and Chapelle, 2024; Arboix-Alió et al., 2025) and increase the risk of injury (Šarabon et al., 2020; Parpa and Michaelides, 2022; Herrera-Amante et al., 2025). Therefore, examining the effects of bilateral asymmetry and muscle co-activation during the two-armed kettlebell swing may provide valuable insights into the exercise’s effectiveness, potentially improving our understanding of performance optimization and injury mechanisms. Thus, the present study aimed to identify differences in muscle co-activation and bilateral asymmetry of shoulder and trunk muscles co-activation during the two-armed kettlebell swing. We hypothesized that bilateral asymmetry in muscle co-activation would be minimal, indicating stable performance supported by symmetrical neuromuscular activation patterns.

## 2 Materials and methods

### 2.1 Subjects and study design

A prior power analysis using G-Power software version 3.1.9.7 (Universität Kiel, Germany) determined that, with six repetitions per participant, a statistical power of 0.80, an alpha level of 0.05, and a medium effect size (f = 0.25), a minimum sample size of 19 participants would be required. To ensure adequate power and account for potential dropouts, 24 participants were recruited for the study (mean age: 23.9 ± 2.5 years; body mass: 82.8 ± 8.0 kg; height: 177.8 ± 6.5 cm). Inclusion criteria required participants to be over 18 years of age and to have a minimum of 5 years of kettlebell training experience. Participants were excluded if they had experienced significant interruptions in their training history (i.e., longer than 6 months) or if they were currently injured or recovering from injuries that could affect performance. All participants provided written informed consent prior to participation. The study protocol was approved by the ethics committee of the hosting institution.

### 2.2 Protocol and data collection

Participants began with a kettlebell-specific warm-up consisting of 5 minutes of submaximal two-armed kettlebell swings. Following the warm-up, each participant performed one set of five repetitions of the two-armed Russian kettlebell swing using a 16-kg kettlebell. The sequence of exercises during the warm-up and experimental trials was kept consistent to ensure standardization. To determine limb dominance, participants were asked which limb they preferred to use during the exercises (Kotsifaki et al., 2022). Swing phase segmentation and timing were determined through video analysis using the Simi Motion Capture System (Simi Reality Motion Analysis, Version 9.0.6), synchronized with the EMG system at a sampling rate of 100 frames per second. The kettlebell swing was divided into four distinct phases: upward propulsion, float, drop, and deceleration. Participants initiated the movement by flexing at the hips during the downward motion, followed by a rapid and forceful hip extension to propel the kettlebell to approximately chest height, in accordance with the Russian swing technique (Meigh et al., 2019). Each participant completed two trials of the two-armed kettlebell swing, with at least five repetitions per trial using the same 16-kg kettlebell (McGill and Marshall, 2012). To avoid fatigue and signal stabilization, a 1-min rest period was provided between trials and the first and last repetitions of each trial were excluded from the analysis. Data from the successful trials (two trials per participant, three repetitions each; total = 144 repetitions) were used to calculate the muscle co-activation index. These values were then used to compute the asymmetry index and conduct the statistical analyses (Zhao et al., 2023).

### 2.3 sEMG signal acquisition and processing

Following SENIAM guidelines, participants’ skin was shaved, lightly abraded, and cleansed with alcohol prior to electrode placement (Hermens et al., 2000). Gel-coated, self-adhesive bipolar surface electrodes (10 mm diameter, silver/silver chloride; SKINTACT FS-RG1/10, Leonhard Lang GmbH, Innsbruck, Austria) were placed with a 2 cm center-to-center distance. Electrodes were positioned on both the dominant and non-dominant sides according to SENIAM recommendations (www.seniam.org) over the following muscles: anterior deltoid (AD), posterior deltoid (PD), erector spinae longissimus (ESL), erector spinae iliocostalis (ESI), rectus abdominis (RA), and external oblique (EO). Surface electromyographic (sEMG) signals were collected using a wireless EMG system (Myon m320RX; Myon, Switzerland) at a sampling frequency of 1000 Hz and a 16-bit A/D resolution. Signal processing was performed using Visual3D software (C-Motion, Germantown, MD, USA). To reduce motion artifacts, a high-pass Butterworth filter at 25 Hz and a low-pass filter at 15 Hz were applied. The signals were then full-wave rectified, and a linear envelope was calculated using the root mean square (RMS) method with a 100 m moving window (Quittmann et al., 2020). EMG amplitudes were subsequently normalized to the isometric maximum voluntary contraction (MVC) of each muscle and expressed as a percentage of MVC (%MVC), and the EMG amplitudes normalized within each trial.

### 2.4 Quantification of co-activation index (CoI)

Muscle co-activation around the dominant arm joints was estimated using the Co-activation Index (CoI) (Equation 1).

$$CoI=\frac{\int_{t_{1}}^{t_{2}}{NEMG}_{antagonist}\left(t\right)\;dt}{\int_{t_{1}}^{t_{2}}\left[{NEMG}_{agonist}+{NEMG}_{antogonist}\right]\left(t\right)dt}\times 100$$ (1)

Where t_1_ and t_2_ represent the beginning and end of each phase, dt denotes the time interval between t_1_ and t_2_, NEMG_antagonist_ denotes the activity of the antagonist muscle, and NEMG_agonist_ denotes the activity of the agonist muscle during the upward propulsion, float, drop, and deceleration phases, separately (Kellis et al., 2003; Oliveira et al., 2017; Akl et al., 2021; Waanders et al., 2021).

### 2.5 Quantification of asymmetry index

The asymmetry Index was calculated following the methodology described by Sheikhi, Letafatkar (Sheikhi et al., 2021), which assesses the degree of bilateral symmetry in muscle activation across the upper limb and trunk muscles (Equation 2).

$$Asymmetry\;Index\;\left(\%\right)=\frac{2\left(\text{dominant}-\text{non}\;\text{dominant}\;\right)\;}{\left(\text{dominant}+\text{non}\;\text{dominant}\;\right)}*100$$ (2)

**FIGURE 1:**
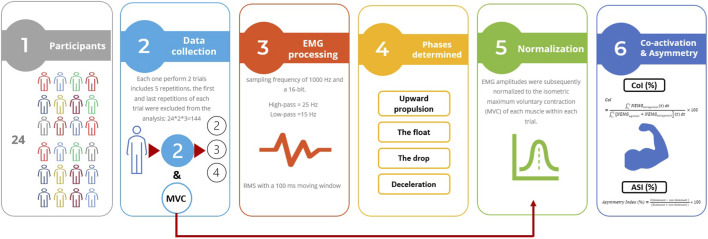
Flow diagram of the data collection and analysis.

An index value of zero indicates perfect symmetry, whereas values greater than zero reflect increasing degrees of asymmetry, with higher values representing more pronounced asymmetry (Whittal et al., 2020). The Asymmetry Index was calculated for the co-activation index of each muscle on both the dominant and non-dominant sides during the two-armed kettlebell swing (Figure 1).

### 2.6 Statistical analysis

Descriptive statistics are reported as means and 95% confidence intervals (mean ± CI). Data normality was assessed using the Shapiro–Wilk test, confirming the suitability for parametric analysis. Within-group differences across movement phases were analyzed using repeated-measures one-way analysis of variance (RM-ANOVA). Sidak *post hoc* tests were applied to compare the means of each variable across the four phases: upward propulsion, float, drop, and deceleration. Effect sizes were calculated using partial eta squared (η^2^_p_) and interpreted as follows: small (η^2^_p_ ≥ 0.01), medium (η^2^_p_ ≥ 0.06), and large (η^2^_p_ ≥ 0.14) (Lakens, 2013). All statistical analyses were conducted using IBM SPSS Statistics version 27 (IBM Corp., Armonk, NY, USA).

## 3 Results

**FIGURE 2:**
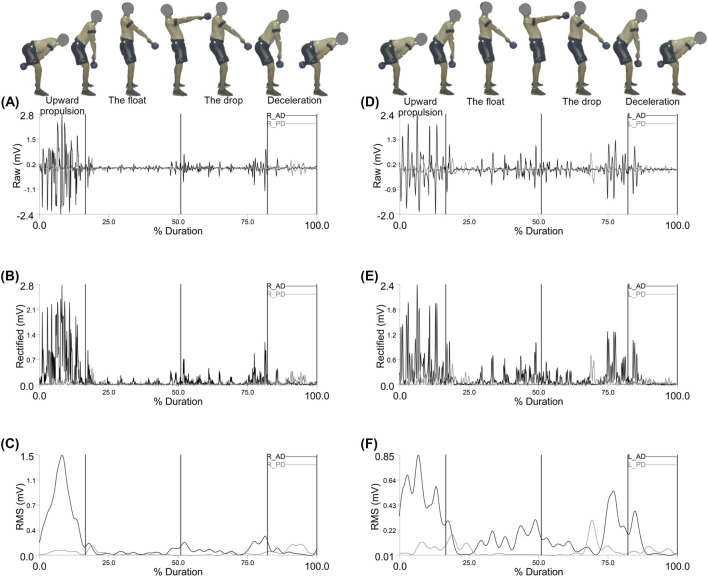
Representative Right anterior deltoid (R_AD) and right posterior deltoid (R_PD) muscle activity: **(A)** EMG Raw; **(B)** EMG Rectified; **(C)** EMG RMS, left anterior deltoid (L_AD) and left posterior deltoid (L_PD) muscle activity: **(D)** EMG Raw; **(E)** EMG Rectified; **(F)** EMG RMS during the double arm swing phases (Upward propulsion, the float, the drop, and deceleration).

Figure 2 shows the electromyographic (EMG) activity of the Right and left anterior deltoid (R_AD) and posterior deltoid (R_PD) shoulder muscles during the double arm swing movement, segmented by phase: Upward Propulsion, Float, Drop, and Deceleration.

**FIGURE 3:**
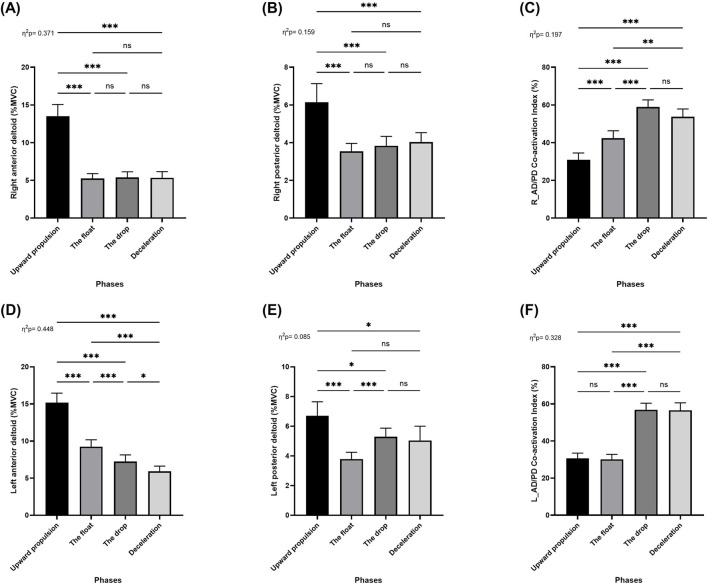
Pairwise comparisons associated with the significant main effects from the RM-ANOVA with mean values and coefficient interval for the normalized EMG (% MVC) of the two-armed muscles of kettlebell exercise during the four phases; **(A)** Right anterior deltoid, **(B)** Right posterior deltoid, **(C)** Right anterior and posterior deltoid co-activation index, **(D)** Left anterior deltoid, **(E)** Left posterior deltoid, and **(F)** Left anterior and posterior deltoid co-activation index during the double arm swing phases (Upward propulsion, the float, the drop, and deceleration). Significant differences for the *post hoc* tests between phases: (***) indicates p < 0.001, (**) indicates p < 0.01, (*) indicates p < 0.05, and (ns) indicates non-significant.

Repeated measures ANOVA (RM-ANOVA) revealed significant main effects of movement phase on muscle activation across deltoid muscles: right anterior deltoid (*F* = 84.25; *p* < 0.001; *η* ^*2*^_*p*_ = 0.37; Figure 3A), right posterior deltoid (*F* = 27.06; *p* < 0.001; *η* ^*2*^_*p*_ = 0.16; Figure 3B), left anterior deltoid (*F* = 115.8; *p* < 0.001; *η* ^*2*^_*p*_ = 0.45; Figure 3D), and left posterior deltoid (*F* = 13.27; *p* < 0.001; *η* ^*2*^_*p*_ = 0.09; Figure 3E). Post hoc analyses indicated that activation of the right anterior and right posterior deltoid significantly decreased from the upward propulsion phase to the float, drop, and deceleration phases (*p* < 0.001), with no significant differences among the float, drop, and deceleration phases (Figures 3A,B). For the left anterior deltoid, activation significantly decreased between the upward propulsion phase and the float, drop, and deceleration phases (*p* < 0.001). Additional reductions were observed between the float and drop (*p* < 0.001), float and deceleration (*p* < 0.001), and drop and deceleration phases (*p* < 0.05; Figure 3D).

In contrast, activation of the left posterior deltoid significantly increased between the upward propulsion and the float (*p* < 0.001), drop (*p* < 0.05), and deceleration phases (*p* < 0.05). A further increase was found between the float and drop phases (*p* < 0.001), while no significant differences were observed between the float and deceleration or the drop and deceleration phases (Figure 3E).

RM-ANOVA also revealed a significant main effect of phase on the shoulder co-activation index for both the right (*F* = 35.12; *p* < 0.001; *η* ^*2*^_*p*_ = 0.20; Figure 3C) and left (*F* = 69.80; *p* < 0.001; *η* ^*2*^_*p*_ = 0.33; Figure 3F) deltoid muscle pairs. Post hoc analysis showed a significant increase in right shoulder co-activation from the upward propulsion phase to the float, drop, and deceleration phases (*p* < 0.001), as well as between the float and drop (*p* < 0.001) and float and deceleration phases (*p* < 0.01), with no significant difference be-tween the drop and deceleration phases (Figure 3C). For the left shoulder co-activation index, significant increases were found between the upward propulsion and drop (*p* < 0.001) and deceleration phases (*p* < 0.001), and between the float and drop (*p* < 0.001) and float and deceleration phases (*p* < 0.01). No significant differences were observed between the upward propulsion and float phases or between the drop and deceleration phases (Figure 3F).

**FIGURE 4:**
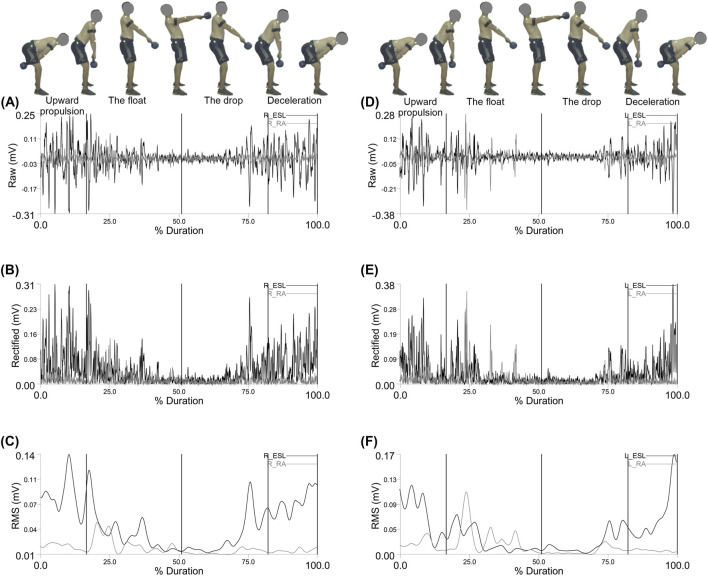
Representative Right erector spinae (longissimus) (R_ESL) and right rectus abdominis (R_RA) muscle activity: **(A)** EMG Raw; **(B)** EMG Rectified; **(C)** EMG RMS, left erector spinae (longissimus) (L_ESL) and left rectus abdominis (L_RA) muscle activity: **(D)** EMG Raw; **(E)** EMG Rectified; **(F)** EMG RMS during the double arm swing phases (Upward propulsion, the float, the drop, and deceleration).

Figure 4 shows the electromyographic (EMG) activity of the Right and left rector spinae (longissimus) (R_ESL) and rectus abdominis (R_RA) trunk muscles during the double arm swing movement, segmented by phase: upward propulsion, float, drop, and deceleration.

**FIGURE 5:**
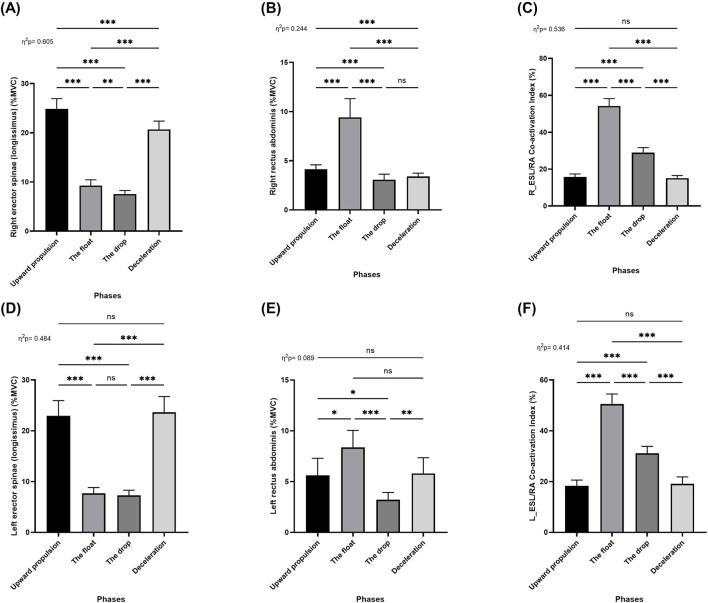
Pairwise comparisons associated with the significant main effects from the RM-ANOVA with mean values and coefficient interval for the normalized EMG (% MVC) of the two-armed muscles of kettlebell exercise during the four phases; **(A)** Right erector spinae (longissimus), **(B)** Right rectus abdominis, **(C)** Right erector spinae (longissimus) and rectus abdominis co-activation index, **(D)** Left erector spinae (longissimus), **(E)** Left rectus abdominis, and **(F)** Left erector spinae (longissimus) and rectus abdominis co-activation index during the double arm swing phases (Upward propulsion, the float, the drop, and deceleration). Significant differences for the *post hoc* tests between phases: (***) indicates p < 0.001, (**) indicates p < 0.01, (*) indicates p < 0.05, and (ns) indicates non-significant.

Repeated measures ANOVA (RM-ANOVA) revealed significant main effects of movement phase on muscle activation for the following muscles: right erector spinae (longissimus) (*F* = 219.1; *p* < 0.001; *η* ^*2*^_*p*_ = 0.61; Figure 5A), right rectus abdominis (*F* = 46.08; *p* < 0.001; *η* ^*2*^_*p*_ = 0.24; Figure 5B), left erector spinae (longissimus) (*F* = 134.3; *p* < 0.001; *η* ^*2*^_*p*_ = 0.48; Figure 5D), and left rectus abdominis (*p* < 0.001; Figure 5E). Post hoc analyses showed that the activation of the right erector spinae (longissimus) significantly decreased from the upward propulsion phase to the float, drop, and deceleration phases (*p* < 0.001). Moreover, activation during the deceleration phase was significantly higher than during both the float and drop phases (*p* < 0.001), and activation during the float phase was significantly higher than the drop phase (*p* < 0.01; Figure 5A). For the right rectus abdominis, muscle activation significantly increased from the upward propulsion to the float phase (*p* < 0.001), followed by a significant decrease from the upward propulsion to the drop and deceleration phases (*p* < 0.001). Additionally, activation during the float phase was significantly greater than during both the drop and deceleration phases (*p* < 0.001), while no significant difference was observed between the drop and deceleration phases (Figure 5B).

Regarding the left erector spinae (longissimus), activation significantly decreased from the upward propulsion phase to the float and drop phases (*p* < 0.001). Activation during the deceleration phase was significantly greater than during both the float and drop phases (*p* < 0.001). However, there were no significant differences between the upward propulsion and deceleration phases, nor between the float and drop phases (Figure 5D). For the left rectus abdominis, activation significantly increased from the upward propulsion to the float phase (*p* < 0.05) and significantly decreased from the upward pro-pulsion to the drop phase (*p* < 0.05). Additionally, activation during the float phase was significantly greater than the drop phase (*p* < 0.001), and activation during the deceleration phase was also significantly greater than the drop phase (*p* < 0.01). No significant differences were found between the upward propulsion and deceleration phases, or between the float and deceleration phases (Figure 5E).

RM-ANOVA also revealed a significant main effect of movement phase on the co-activation index between the erector spinae (longissimus) and rectus abdominis muscles on both the right (*F* = 165.1; *p* < 0.001; *η* ^*2*^_*p*_ = 0.54; Figure 5C) and left sides (*F* = 100.08; *p* < 0.001; *η* ^*2*^_*p*_ = 0.41; Figure 5F). Post hoc analyses indicated that co-activation significantly increased from the upward propulsion to the float phase (*p* < 0.001), followed by a significant decrease from the float to the drop and deceleration phases (*p* < 0.001), as well as between the drop and deceleration phases (*p* < 0.001). No significant differences were observed between the upward propulsion and deceleration phases (Figures 5C,F).

**FIGURE 6:**
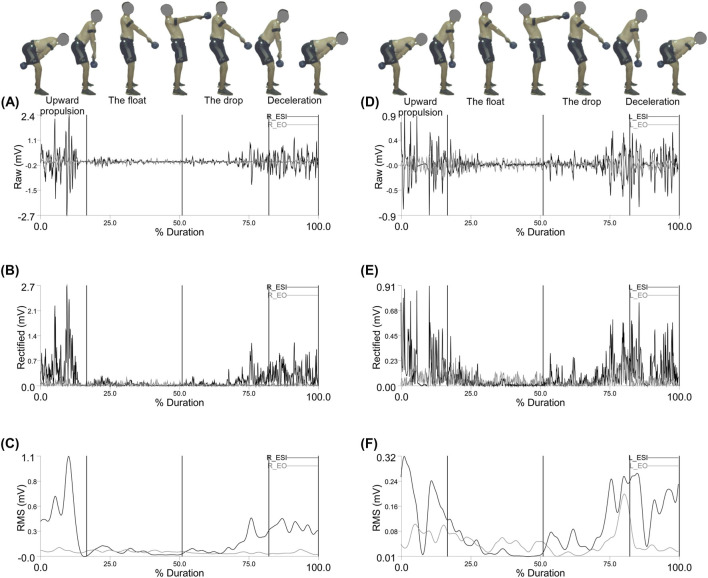
Representative Right erector spinae (iliocostalis) (R_ESI) and right external oblique (R_EO) muscle activity: **(A)** EMG Raw; **(B)** EMG Rectified; **(C)** EMG RMS, left erector spinae (iliocostalis) (L_ESI) and left external oblique (L_EO) muscle activity: **(D)** EMG Raw; **(E)** EMG Rectified; **(F)** EMG RMS during the double arm swing phases (Upward propulsion, the float, the drop, and deceleration).

Figure 6 shows the electromyographic (EMG) activity of the Right and left erector spinae (iliocostalis) (R_ESI) and external oblique (R_EO) trunk muscles during the double arm swing movement, segmented by phase: upward propulsion, float, drop, and deceleration.

**FIGURE 7:**
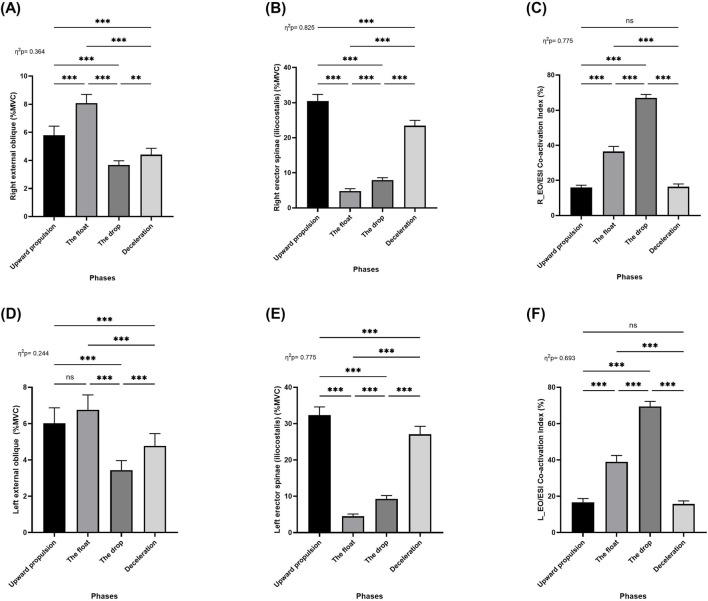
Pairwise comparisons associated with the significant main effects from the RM-ANOVA with mean values and coefficient interval for the normalized EMG (% MVC) of the two-armed muscles of kettlebell exercise during the four phases; **(A)** Right external oblique, **(B)** Right erector spinae (iliocostalis), **(C)** Right external oblique and erector spinae (iliocostalis) co-activation index, **(D)** Left external oblique, **(E)** Left erector spinae (iliocostalis), and **(F)** Left external oblique and erector spinae (iliocostalis) co-activation index during the double arm swing phases (Upward propulsion, the float, the drop, and deceleration). Significant differences for the *post hoc* tests between phases: (***) indicates p < 0.001, (**) indicates p < 0.01, (*) indicates p < 0.05, and (ns) indicates non-significant.

Repeated measures ANOVA (RM-ANOVA) revealed significant main effects of movement phase on muscle activation for the following muscles: right external oblique (*F* = 81.93; *p* < 0.001; *η* ^*2*^_*p*_ = 0.36; Figure 7A), right erector spinae (iliocostalis) (*F* = 672.7; *p* < 0.001; *η* ^*2*^_*p*_ = 0.83; Figure 7B), left external oblique (*F* = 46.16; *p* < 0.001; *η* ^*2*^_*p*_ = 0.24; Figure 7D), and left erector spinae (iliocostalis) (*F* = 491.7; *p* < 0.001; *η* ^*2*^_*p*_ = 0.78; Figure 7E). Post hoc analyses showed that the activation of the right external oblique, muscle activation significantly increased from the upward propulsion to the float phase (*p* < 0.001), followed by a significant decrease from the upward propulsion to the drop and deceleration phases (*p* < 0.001). Additionally, activation during the float phase was significantly greater than during both the drop and deceleration phases (*p* < 0.001), while a significant decreased was observed between the drop and deceleration phases (*p* < 0.01; Figure 7B). For the right erector spinae (iliocostalis) significantly decreased from the upward propulsion phase to the float, drop, and deceleration phases (*p* < 0.001). Moreover, activation during the deceleration phase was significantly higher than during both the float and drop phases (*p* < 0.001), and activation during the float phase was significantly higher than the drop phase (*p* < 0.001; Figure 7B).

Regarding the left external oblique, activation significantly decreased from the upward propulsion to the drop and deceleration phases (*p* < 0.001). Additionally, activation during the float phase was significantly greater than the drop and deceleration phases (*p* < 0.001), and activation during the deceleration phase was also significantly greater than the drop phase (*p* < 0.001). No significant differences were found between the upward propulsion and the float phases (Figure 7D). For the left erector spinae (iliocostalis), activation significantly decreased from the upward propulsion phase to the float, drop, and deceleration phases (*p* < 0.001). Activation during the deceleration phase was significantly greater than during both the float and drop phases (*p* < 0.001). Additionally, there were the drop phase significantly higher than the float phase (*p* < 0.001; Figure 7E).

RM-ANOVA also revealed a significant main effect of movement phase on the co-activation index between the erector spinae (iliocostalis) and external oblique muscles on both the right (*F* = 491.6; *p* < 0.001; *η* ^*2*^_*p*_ = 0.78; Figure 7C) and left sides (*F* = 322.3; *p* < 0.001; *η* ^*2*^_*p*_ = 0.69; Figure 7F). Post hoc analyses indicated that co-activation significantly increased from the upward propulsion, the float, and drop phases (*p* < 0.001), followed by a significant decrease from the drop and deceleration phases (*p* < 0.001). No significant differences were observed between the upward propulsion and deceleration phases (Figures 7C,F).

**FIGURE 8:**
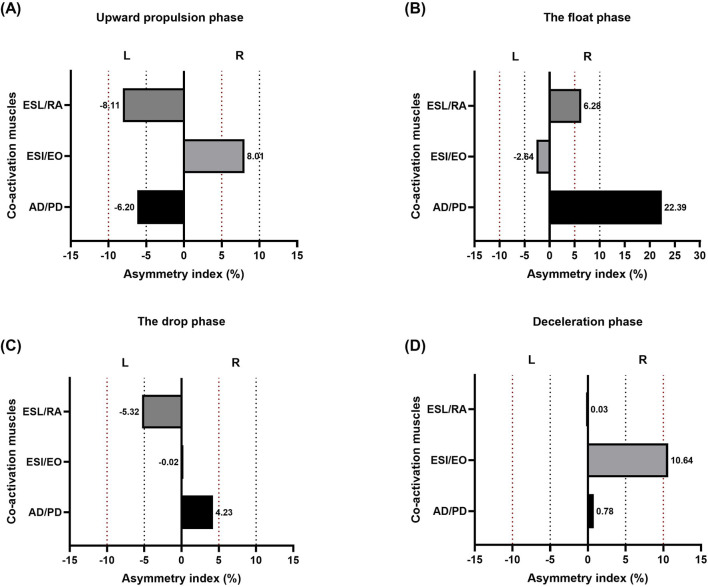
Muscle asymmetry data (%) during the two-armed kettlebell swing across four movement phases: **(A)** upward propulsion, **(B)** float, **(C)** drop, and **(D)** deceleration. Bars extending to the right indicate asymmetry favoring the right side, while bars extending to the left indicate asymmetry favoring the left side. Dashed lines represent asymmetric thresholds: red for 10% and black for 5%, on both the right (R) and left (L) sides.

Figure 8 provides asymmetrical indices for individual muscle co-activation during each phase. During upward propulsion, asymmetry indices were AD/PD muscles co-activation (−6.20%), ESI/EO muscles co-activation (8.01%), and ESL/RA muscles co-activation (−8.11%) (Figure 8A). During the float phase, asymmetry indices were AD/PD muscles co-activation (22.39%), ESI/EO muscles co-activation (−2.64%), and ESL/RA muscles co-activation (6.28%) (Figure 8B). During the drop phase, asymmetry indices were AD/PD muscles co-activation (4.23%), ESI/EO muscles co-activation (−0.02%), and ESL/RA muscles co-activation (−5.32%) (Figure 8C). During deceleration asymmetry indices were AD/PD muscles co-activation (0.78%), ESI/EO muscles co-activation (10.64%), and ESL/RA muscles co-activation (0.03%) (Figure 8D).

## 4 Discussion

The aim of this study was to identify differences in muscle co-activation and bilateral asymmetry of the shoulder and trunk muscles co-activation during the two-armed kettlebell swing. To the best of our knowledge, this is the first study to investigate co-activation asymmetry in this specific exercise. This approach offers valuable insights into the effectiveness of the technique and enhances our understanding of performance stability through symmetrical neuromuscular activation.

The findings indicate that deltoid muscle activation differs markedly across the phases of the two-armed kettlebell swing. In particular, these muscles are crucial during the initial upward phase, where they contribute substantially to kettlebell acceleration, consistent with Salem et al. (2021). Notably, the upward propulsion phase elicited the highest activation in the anterior deltoids bilaterally, which subsequently declined during the float, drop, and deceleration phases. This pattern aligns with the biomechanical demands of propulsion, where the anterior deltoid plays a critical role in shoulder flexion and stabilization against the upward momentum of the kettlebell. The gradual decline in activation across subsequent phases likely reflects the reduced muscular demand as the kettlebell transitions into a ballistic, passive trajectory (Padovan et al., 2024). Both the right anterior and posterior deltoids followed similar activation patterns, with no significant differences observed among the float, drop, and deceleration phases. This suggests a consistent stabilizing role once the primary forceful effort is complete. Interestingly, the left anterior deltoid displayed a more progressive decline in activation, potentially indicating side-specific neuromuscular strategies or mild asymmetries in motor control (Padovan et al., 2024). In contrast, the left posterior deltoid exhibited increased activation from the upward propulsion through to the later phases, possibly reflecting a compensatory stabilizing function during float and drop, assisting in posterior shoulder stability as the arm extends and decelerates (Padovan et al., 2024). The absence of this trend in the right posterior deltoid may suggest dominance-related differences or asymmetrical movement mechanics.

Analysis of the shoulder co-activation index revealed significant increases on both sides during the float, drop, and deceleration phases, indicating a transition from prime mover dominance to enhanced joint stabilization (Mari et al., 2014; Lee et al., 2017). The elevated co-activation in these phases likely represents a protective strategy to preserve shoulder stability under high-speed, dynamic conditions, particularly as the kettlebell descends and changes direction. Notably, the more pronounced increase on the left side corresponds with the increased posterior deltoid activity, supporting the presence of asymmetrical neuromuscular engagement in complex ballistic tasks. Collectively, these results highlight the phase-dependent nature of deltoid engagement and shoulder control. The anterior deltoids dominate during power generation, while the posterior deltoids and overall co-activation increase during the later phases to modulate momentum and stabilize the shoulder (Garcia-Vicencio et al., 2024). These insights deepen our understanding of shoulder muscle coordination in ballistic tasks and have implications for rehabilitation, injury prevention, and training design, particularly when addressing muscle imbalances or neuromuscular asymmetries.

The present results demonstrate distinct phase-dependent activation patterns in trunk muscles, underscoring the dynamic contributions of the erector spinae (longissimus) and rectus abdominis during the kettlebell swing. These muscles are particularly important for trunk extension in the upward phase and for maintaining spinal stability under load to prevent spinal twisting (Andersen et al., 2016). Moreover, activation of the core extensors in response to flexion moments further reinforces their critical stabilizing function (Brown and Potvin, 2005). Significant variations across phases underscore how trunk musculature adapts to shifting mechanical demands related to force generation, stabilization, and impact control. The erector spinae on both sides exhibited peak activation during the upward propulsion phase, underscoring their key role in lumbar extension and postural maintenance. The decline in activation during the float and drop phases reflects a transition from active effort to passive ballistic movement. A notable increase in activation during deceleration, particularly on the right, may reflect renewed muscular engagement for trunk flexion control and impact absorption (Abuwarda and Akl, 2024; Padovan et al., 2024). The asymmetrical pattern with more sustained right-side activity may indicate mild lateral imbalances in trunk stabilization. The rectus abdominis demonstrated a different temporal pattern. Both sides increased in activity during the float phase, possibly to maintain anterior trunk stability as the posterior chain disengages. This finding supports the notion that the rectus abdominis compensates for reduced activation of the spinal extensor muscles (Le et al., 2016). The subsequent decline during the drop and deceleration phases likely reflects a re-engagement of the posterior musculature as the trunk moves into flexion under the influence of gravity.

Phase-specific changes in co-activation between erector spinae and rectus abdominis further support this interpretation. Increased co-activation during the float phase may represent a bracing mechanism to maintain trunk rigidity (Beneck et al., 2016). The drop in co-activation during the drop and deceleration phases likely allows for greater flexibility and energy absorption. The observed symmetry in co-activation across sides suggests a well-coordinated neuromuscular strategy for core stabilization. Together, these results highlight the interplay between spinal extensors and abdominal muscles in response to the demands of ballistic movement. The shift from posterior to anterior engagement and back, coupled with strategic co-activation, reinforces the importance of balanced core training for performance and injury prevention.

Additionally, the findings show that the activation of the external obliques and erector spinae (iliocostalis) varies considerably across different movement phases, reflecting the complex neuromuscular coordination required for trunk stabilization during kettlebell swings. He relatively small side-to-side asymmetry observed may help reduce injury risk while enhancing training efficiency (Zhang et al., 2021; Stagi et al., 2023). The erector spinae (iliocostalis) on both sides showed peak activation during the upward propulsion phase, aligning with their role in maintaining lumbar extension under load (Beneck et al., 2016). As the swing progresses into float and drop phases, activation decreases significantly, consistent with the reduced need for spinal extension. The rise in activity during the deceleration phase, particularly on the left, suggests these muscles re-engage to resist trunk flexion and manage momentum. The external obliques demonstrated asymmetrical yet phase-dependent activation. The right side peaked during the float phase and declined thereafter, potentially reflecting its role in rotational stability and anti-lateral flexion. In contrast, the left external oblique showed sustained activation into the deceleration phase, indicating a possible dominance or compensatory stabilization strategy. Such patterns highlight the relevance of side-specific trunk engagement, especially during high-speed tasks (Salem et al., 2021; Padovan et al., 2024).

The co-activation index between the erector spinae and external oblique muscles also varied significantly with phase. Co-activation was highest during the float and drop phases, likely representing a deliberate stabilization strategy during dynamic transitions. This bracing enhances spinal stiffness and helps maintain control under variable forces. The subsequent drop in co-activation during deceleration suggests controlled relaxation to accommodate trunk flexion and absorb load. The similarity between the deceleration and upward propulsion phases in co-activation patterns may point to shared stabilization requirements at these critical transitional points (Lee et al., 2017; Pincivero et al., 2019). These findings underscore the importance of trunk muscle coordination in ensuring control throughout all swing phases and reinforce the role of core musculature in managing ballistic load transfer.

Finally, the analysis of asymmetry indices in muscle co-activation offers novel insights into bilateral neuromuscular coordination during kettlebell swings. While some asymmetry is expected in complex motor tasks, the magnitude and direction of asymmetry can inform us about motor strategies, dominance, or compensations (Herrera-Amante et al., 2025). During the upward propulsion phase, asymmetry indices were relatively low across all muscle groups (ranging from −8.11% to +8.01%), suggesting highly coordinated bilateral muscle recruitment to ensure symmetrical force generation. Minor asymmetries likely reflect individual movement variability or limb dominance (Foley et al., 2025). In contrast, the float phase showed the greatest asymmetry, particularly in the AD/PD muscle pair (22.39%), suggesting a shift toward less synchronized bilateral coordination during this passive phase. As the kettlebell is in ballistic flight, the body may rely more on intrinsic stabilization mechanisms and habitual movement patterns, making this phase more susceptible to side-dominant behavior or imbalance. By the drop phase, asymmetry indices decreased across all muscles, indicating a return to more synchronized bilateral control, likely driven by the need to prepare for impact absorption. The deceleration phase also showed low asymmetry, with most values close to zero, further suggesting re-established coordination for stabilization before initiating the next repetition. However, the ESI/EO pair showed moderate asymmetry (10.64%), indicating persistent side bias in trunk stabilization.

Although this study yielded meaningful findings, its small sample size restricts generalizability, highlighting the need for larger cohorts in future research. The analysis was limited to trunk and upper-limb muscles, excluding lower-limb activity that is fundamental to kettlebell swings; incorporating these muscles would provide a more complete perspective on exercise demands. Furthermore, investigating the effects of varying kettlebell loads and swing techniques could clarify how factors such as external load, age, training history, and muscle co-activation asymmetry interact.

Overall, these patterns suggest that neuromuscular asymmetry fluctuates by phase, with the greatest variability occurring during float, where external demands are lowest and intrinsic strategies dominate. Lower asymmetry in propulsion, drop, and deceleration phases likely reflects the body’s need for more symmetrical and controlled effort in response to mechanical demands (Arboix-Alió et al., 2025). These insights are particularly valuable for training, injury prevention, and rehabilitation, where recognizing phase-specific asymmetry can inform interventions. Targeted strategies may be especially important in reducing injury risk associated with asymmetric loading in ballistic movements.

## 5 Conclusion

This study investigated muscle co-activation and bilateral asymmetry in trunk and shoulder musculature across the movement phases of the two-armed kettlebell swing. The findings revealed clear phase-dependent patterns of muscle activation and co-activation, highlighting the dynamic neuromuscular strategies required for both force generation and joint stabilization. Anterior deltoid and spinal extensor muscles dominated during the propulsion phase, while increased co-activation and posterior muscle engagement during the float, drop, and deceleration phases supported controlled descent and joint stability. Importantly, while some asymmetry in muscle co-activation was observed, particularly during the float phase, overall asymmetry levels remained low during the more mechanically demanding phases (propulsion, drop, and deceleration). This supports our hypothesis that bilateral asymmetry in muscle co-activation is minimal during key points of the swing, reflecting coordinated and symmetrical neuromuscular control. These results emphasize the effectiveness of the kettlebell swing in promoting balanced activation of trunk and shoulder muscles and provide valuable insights for training, injury prevention, and rehabilitation programs focused on improving movement symmetry and core stability.

## Data Availability

The raw data supporting the conclusions of this article will be made available by the authors, without undue reservation.
